# Angular orientation between the cores of iron oxide nanoclusters controls their magneto–optical properties and magnetic heating functions

**DOI:** 10.1038/s42004-022-00787-0

**Published:** 2022-12-02

**Authors:** Enzo Bertuit, Nicolas Menguy, Claire Wilhelm, Anne-Laure Rollet, Ali Abou-Hassan

**Affiliations:** 1 Sorbonne Université, UMR CNRS 8234, PHysico-chimie des Électrolytes et Nanosystèmes InterfaciauX (PHENIX), F-75005 Paris, France; 2 Sorbonne Université, UMR 7590 CNRS—Sorbonne Université—IRD-MNHN, Institut de Minéralogie, de Physique des Matériaux et de Cosmochimie (IMPMC), Case 115, 4 Place Jussieu, 75252 Cedex 5 Paris, France; 3 PSL Research University—Sorbonne Université—CNRS, UMR168, Laboratoire Physico Chimie Curie, Institut Curie, 75005 Paris, France; 4 Institut Universitaire de France (IUF), 75231 Cedex 05 Paris, France

**Keywords:** Nanoscale materials, Physical chemistry

## Abstract

Oriented attachment of nanobricks into hierarchical multi-scale structures such as inorganic nanoclusters is one of the crystallization mechanisms that has revolutionized the field of nano and materials science. Herein, we show that the mosaicity, which measures the misalignment of crystal plane orientation between the nanobricks, governs their magneto-optical properties as well as the magnetic heating functions of iron oxide nanoclusters. Thanks to high-temperature and time-resolved millifluidic, we were able to isolate and characterize (structure, properties, function) the different intermediates involved in the diverse steps of the nanocluster’s formation, to propose a detailed dynamical mechanism of their formation and establish a clear correlation between changes in mosaicity at the nanoscale and their resulting physical properties. Finally, we demonstrate that their magneto-optical properties can be described using simple molecular theories.

## Abstract (web summary)

Oriented attachment of nanoscale building blocks into hierarchical structures offers vast opportunities to engineer nanomaterial structure-property relationships. Here, the misalignment of crystal plane orientations between iron oxide nanoclusters is shown to govern their magneto–optical properties and magnetic heating functions.

## Introduction

Magnetic nanoparticles have been particularly studied during the last decades, mainly because of their interesting magnetic properties and the versatility of their surface functionalization^1–4^. Indeed, their high saturation magnetization values and their superparamagnetic character can be used for many applications including magnetic resonance imaging^5^, sensing^6^, drug delivery^7^, for thermal therapies by magnetic hyperthermia^8^ or as more recently for their optical properties in photothermal applications^9–11^. In the particular case of nanoclusters, the interactions between the “bricks” forming the nanoassembly can be the source of synergistic effects, making the physico-chemical properties of these objects even more particular.

In the field of materials science, the concept of colloidal molecules^12–14^, as well as the discovery of oriented attachment mechanisms^15–17^, have paved the way to the engineering and rationalization of a large number of structures including assemblies and clusters of different geometries and length scales^18–22^. For example, the polyol process has shown its versatility for the preparation of many types of aggregates with controlled morphologies including iron oxide nanoclusters^23–25^. Magnetic multi-core iron oxide nanoparticles (NPs), or nanoflowers, are quasi-monocrystalline nanostructures formed by aggregation and oriented attachment of their tiny cores. Lately, these mesocrystals have received particular attention due to their unique magnetic and optical properties especially for biomedical applications, making them one of the most efficient available nanoheaters for thermal therapies in magnetic hyperthermia and NIR-I-II photothermia^10,24,26–28^. Although the formation of such clusters was proposed to occur through green rust intermediates^28,29^, to date a dynamic mechanistic study on their formation correlating their structure changes with time to their physical properties in real high temperature conditions is still missing.

In this work, we explore using a multi-scale approach combining millifluidics, high-resolution transmission electron microscopy (HR-TEM), and physical characterizations (magnetic and optical) the formation of iron oxide nanoclusters with time. Insights on the mechanisms involved in nanocluster formation are elucidated thanks to millifluidics which enables a 30 s temporal resolution and an easy isolation of different formation intermediates. We show particularly how the different growth stages impact their collective magnetic and optical properties. Remarkably, we prove how little changes in the nanocluster fine structure during their growth, as reflected by mosaicity measurements, modulate their physical properties. Finally, we demonstrate that molecular theories, i.e., the magnetic exchange coupling theory for molecular magnets^30^ and the excitonic theory^31^ for absorption of molecular H-aggregates can be extended to the level of the nanostructures to explain their magnetic and optical features.

## Results and discussion

### Chasing the formation of nanoflowers by time-resolved millifluidics

**Fig. 1:**
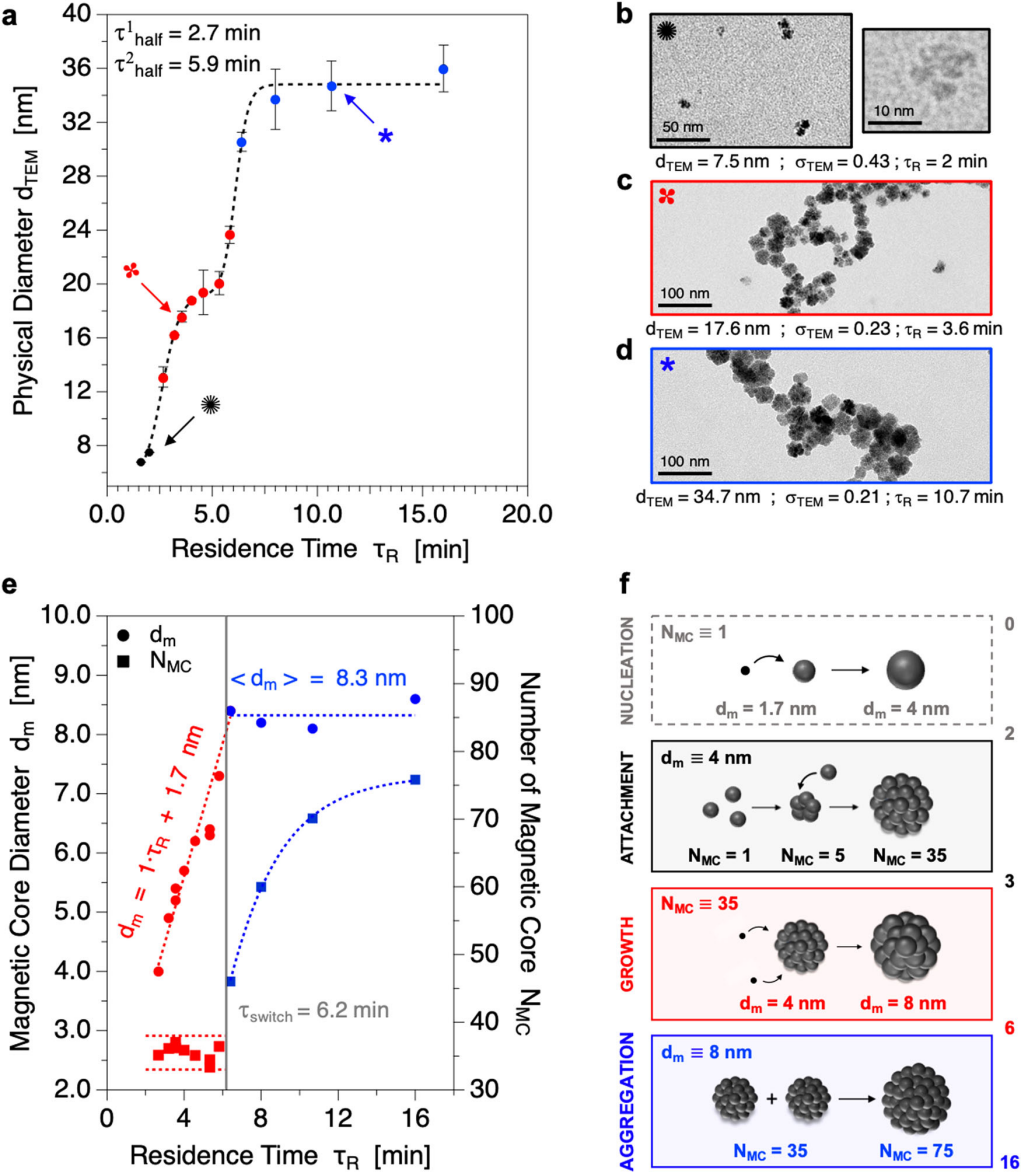
Nanoscale investigation of the mechanisms involved in NFs formation. **a** Evolution of the physical diameter observed by TEM with the residence time in the system (error bars correspond to the standard deviations on diameters for 2 or 3 repeated syntheses). The residence time inside the millifluidic channel was calculated by τ_R_ = Q/V with Q is the total flow rate and V the total volume of the channel. **b**–**d** Representative TEM micrographs of 3 samples obtained respectively for residence times of 2.0, 3.6, and 10.7 min, indicated by arrows in (**a**). **e** Evolution of the core diameter and the number of magnetic cores within the NFs assemblies with the residence time. **f** Schematic representation of the mechanisms leading to NFs morphological sequential evolutions (small black dots: iron(II) and/or iron(III) molecular precursors, dark gray balls: iron oxide cores).

To understand the formation of the nanoflowers (NFs) at the nanoscale, time-resolved kinetic studies were possible due to a home-made high temperature continuous flow millifluidic device previously developed and described by our group^32^ (see also Supplementary Fig. 1). In addition to high chemical yields and an easy scaling up of the synthesis, this system offers an excellent temporal resolution of about 30 s. As shown in Fig. 1a, the evolution of the physical diameter (d_TEM_) with residence times (τ_R_) displays three distinct regimes (black, red, blue) separated respectively by characteristic exponential time constants of τ^1^_half_ = 2.7 min and τ^2^_half_ = 5.9 min. In the first phase, isolated spherical cores and small flower-like particles with approximately 5 cores of 4 nm are observed by transmission electron microscopy (TEM) while the presence of well-defined NFs is evidenced in the second and third phases (Fig. 1b–d, see also Supplementary Figs. 2–4). The d_TEM_ = *f*(τ_R_) curve progression suggests that those three steps are separated by two different activation energy barriers corresponding to the transformation of flower-like structures into well-defined NFs and after of small NFs into bigger ones, respectively. To gain more insights into the NFs formation mechanism, the size of magnetic cores (d_m_) and the number of cores (N_MC_) per flower-like assembly are determined by VSM (see also Supplementary Fig. 5) for residence times corresponding to the second and third steps (i.e., between 3 min and 16 min, Fig. 1e). Again, two different behaviors are evidenced with a characteristic switch time of τ_switch_ = 6.2 min, very close to the characteristic time of τ^1^_half_ = 5.9 min determined previously. Firstly, the magnetic core size increases from 4 nm to 8 nm while the number of cores remains constant around N_MC_ = 35. The linear evolution observed in the first step also suggests that nuclei formed at early times are about 1.7 nm in diameter. Then, the magnetic core size reaches a plateau for a value of d_m_ = 8.3 nm, while the number of cores increases exponentially from approximately N_MC_ = 45 to N_MC_ = 75. In a consistent way, TEM observations show an increase in the NFs diameter. Taken together, these results evidence the presence of a nucleation step before 3 min, followed by a growth step between 3 and 6 min with a final aggregation step for residence times higher than 6 min. To explore the aggregation mechanism into bigger NFs, SAXS was performed on the crude final products in polyol solvents collected at the output of the millifluidic system for each residence time (see Supplementary Fig. 6). A characteristic NF-NF interaction peak is evidenced and corresponds to a NF-NF distance similar to the NFs diameter obtained from TEM. Interestingly, no significant peak is observed in the *q*-range of core sizes (4–8 nm) indicating that no or undetectable isolated cores are present in the crude products. Therefore, the increase in the number of magnetic cores observed in the last stage of NFs formation can be explained by the attachment of NFs to neighbor NFs giving rise to a higher number of cores with a constant size of 8 nm. According to TEM, VSM and SAXS data, we propose the following mechanism for NFs formation (Fig. 1f). At early residence times, 1.7 nm spherical nuclei are formed (as extrapolated on d_m_ = *f*(τ_R_) curve) from molecular precursors reaching a final size of 4 nm. At about τ_R_ = 3 min, primary poorly-defined flower-like structures composed of 5 cores of 4 nm are next formed followed by a very fast attachment of isolated cores to create 35-cores structures. Afterward, the number of cores remains constant (3 $\le$ τ_R_ $\le$ 6 min) and the dissolution of molecular precursors into the nanostructures enables the growth of each core from 4 nm to 8 nm. Finally, when the core size reaches the critical value of about 8 nm, core growth stops and NFs start to attach to other NFs, yielding bigger NFs with a higher number of 8 nm cores (τ_R_ $\ge$ 6 min). By analogy with molecular magnets, this mechanistic study suggests that superparamagnetic flower-like nanoclusters may be considered as colloidal molecules formed by the assembly of several cores acting as magnetic atom centers. From now on, as we have shown that the syntheses in the millifluidic device are highly reproducible (very small error bars in Fig. 1a), we will assume that all the measured properties should be reproducible because both magnetic and optical properties strongly depend on the size and composition of the nanoparticles.

### Magneto-optical properties are time-dependent

**Fig. 2:**
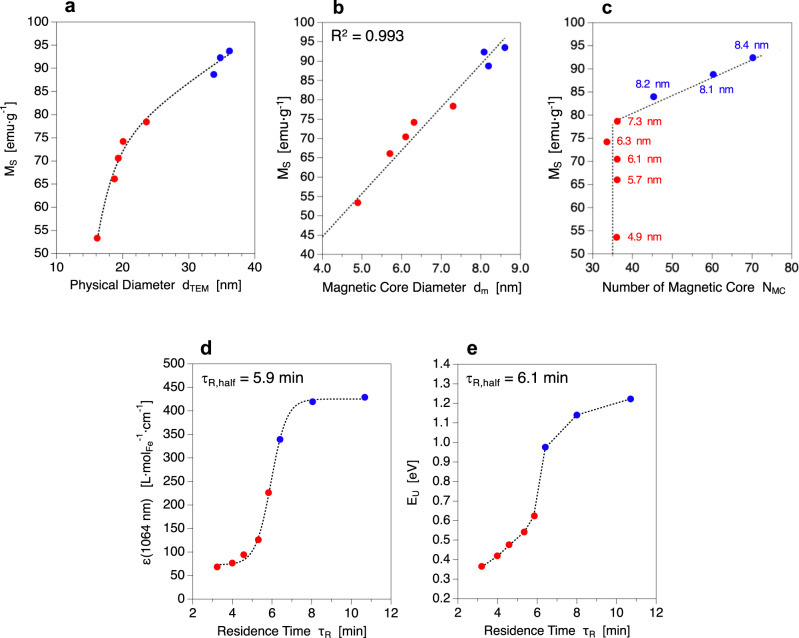
Evolution of NFs static magnetic properties and optical characteristics with nanostructural features and residence time. **a**–**c** Saturation magnetization dependency on the physical diameter, magnetic core diameter and number of magnetic cores, respectively. **d**, **e** Evolution of the extinction coefficient and Urbach energy with residence time, respectively. Please note that the sizes on Fig. 2c correspond to the magnetic core diameters.

The next logical step is to investigate how these nanostructural transformations translate into changes in the NFs physical features, mainly their magnetic and optical properties (Fig. 2). Regarding magnetism, the saturation magnetization (M_S_) of the NFs is found to increase with their physical diameter to reach values up to 90–95 emu/g (Fig. 2a, see also Supplementary Fig. 7) during the final aggregation stage, in good agreement with previously reported values for iron oxide NFs^33^. Surprisingly, the saturation magnetization depends linearly on the size of the magnetic cores (Fig. 2b) and not on the volume of the NFs. However, it is well established that M_S_ increases with exchange couplings and therefore with the alignment between the crystalline planes of the different cores in the mesocrystal^34,35^. Thus, such a result strongly suggests a progressive orientation of the magnetic cores during their growth. Besides, M_S_ increases with N_MC_ following two distinct rates (Fig. 2c). Firstly, during the growth stage where the number of magnetic cores is constant (N_MC_ $\equiv$ 35) and d_m_ grows from 4 nm to 8 nm, an increase independent from N_MC_ is observed. Then, a lower slope of about 0.3 emu∙g^−1^∙core^−1^ is evidenced when N_MC_ increases up to 75 with a constant magnetic core size of 8 nm. Interestingly, the switch from one rate to the other matches perfectly with the switch time highlighted by TEM (τ_R_ = 5.8 min) and can be explained by the appearance of more dipolar effects that tend to decrease the rise of M_S_ values when N_MC_ increases, as predicted by numerical simulations^36^. Taken together, these results suggest that the magnetic macrospin of the NFs does not result in the simple sum of each core magnetic spin but is a more complex function of both the core size and core number. The variation of NFs electronic band structure during their formation is tracked by studying their optical properties. XANES at Fe K-edge analyses^37–39^ (see Supplementary Fig. 8) show that all samples have the stoichiometry of pure magnetite (R = Fe^II^/Fe^III^ = 0.5) with an average value of <R> = 0.48 $\pm$ 0.01. Consequently, the intervalence charge transfer (IVCT)^40^ between Fe^3+^ and Fe^2+^ laying in the NIR-II window is used as a fingerprint of electronic effects. The extinction coefficient in the NIR-II region (ε_1064_) is deduced from UV-Vis-NIR absorption spectra (see Supplementary Figs. 9 and 10) and is found to follow a sigmoid function of residence time, again with a characteristic time τ_R,half_ = 5.9 min (Fig. 2d). As ε_1064_ variations cannot be ascribed to differences in stoichiometry, the band structure of the semi-conductor NFs is explored and characterized by direct band-gap energy (E_g_^dir^) and defect-characteristic^41,42^ Urbach energy (E_U_). Consistently with previously reported values^43^, the same direct band-gap energy is found for all NFs with an average value of <E_g_^dir^> = 3.7 $\pm$ 0.1 eV (see Supplementary Fig. 11). The Urbach energy, which translates and quantifies the presence of all type of defects (chemical composition, structural/crystalline disorders) in semi-conductors, is then calculated from UV-Vis-NIR absorption spectra (see Supplementary Fig. 12) according to Urbach theory^44^. A comparable sigmoidal evolution of E_U_ values with residence time is observed when compared to ε_1064_ variations with a similar characteristic time of τ_R,half_ = 6.1 min (Fig. 2e). As E_U_ is inversely proportional to the number of defects, these results strongly suggest that the increase in ε_1064_ is due to a diminution of defect concentration within the band structure of the NFs.

### Evolution of magnetic spin relaxation processes with time

**Fig. 3:**
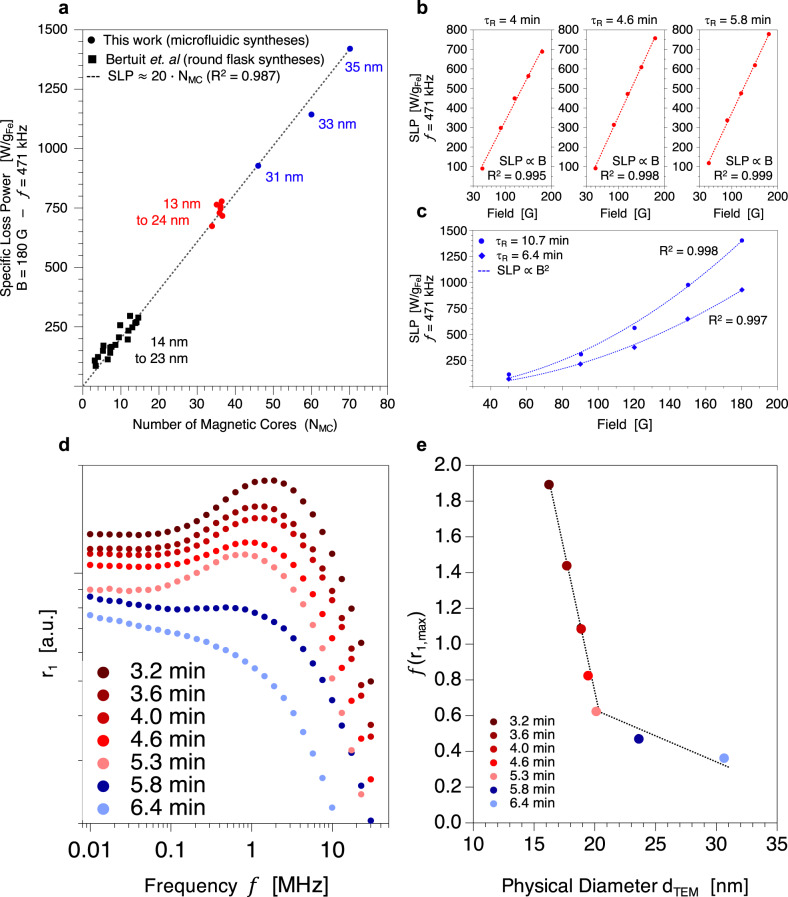
NFs dynamic magnetic properties: magnetic hyperthermia and longitudinal relaxivity profiles. **a** SLP as a function of number of magnetic cores and comparison with previous work for an AMF of 180 G and 471 kHz. **b**, **c** SLP as a function of AMF strength at a fixed frequency of 471 kHz for residence times of 4, 4.6, 5.8 min and 6.4, 10.7 min. **d** r_1_ longitudinal relaxivity profiles for different residence times. **e** Frequency position *f* of the maximum r_1,max_ as a function of physical diameter.

To better understand how the core transformations impact the magnetic properties of the whole NFs assemblies, dynamic magnetic properties of the NFs are next investigated. In the case of nanoclusters, it is established that magnetic interactions between each “brick” of the cluster strongly impact their resulting efficiency in magnetic hyperthermia (MHT)^27^. For iron oxide NF-like nanoclusters, the presence of exchange couplings between the magnetic cores highly enhances the specific loss power (SLP) when compared to single-core structures such as nanospheres^25,26^ or nanocubes^45–47^. Figure 3 shows MHT performances of the NFs under different alternative magnetic field (AMF) excitations which remain below the Atkinson-Brezovich safety limit (see also Supplementary Fig. 13)^48^. For a given AMF frequency and amplitude, SLP increases with the number of magnetic cores composing the NFs rather than with the physical diameter of the assembly (Fig. 3a). When comparing the results for NFs synthetized in the milifluidic device (circles) with those obtained for round-flask NFs syntheses (squares, Bertuit et al.^10^), it is found that the SLP of iron oxide NFs is proportional to N_MC_ for a given AMF. Such a linear correlation can be explained by an increase of the exchange couplings, that are an increasing function of N_MC_ in the case of flower-like nanoclusters^24^. The same tendency was reported by Storozhuk et al.^49^ who showed an increase in the SLP with the number of cores thanks to a seeded-growth synthesis enabling the obtention of nanoassemblies with various number of cores. Even if the number of cores was not determined in this study, the authors clearly show an increase in the SLP after the first and the second step of seeding associated to an increase in the number of cores evidenced by TEM observations. In our case, high SLP $\ge$ 700 W/g values are reached, due to the high number of cores forming the nanossemblies obtained in the millifluidic device. NFs between 13 nm and 24 nm (3 min $\le$ τ_R_ $\le$ 6 min, N_MC_ = 35) present intrinsic loss power (ILP) of 8.2 $\pm$ 0.3 nH∙m^2^∙kg_Fe_^−1^, which are higher than most of the previously reported values for iron oxide NFs of similar sizes. Hugounenq et al.^24^ reported ILP up to 6.4 nH∙m^2^∙kg_Fe_^−1^ for 24 nm NFs obtained by 48 h polyol synthesis. Herein, for residence times of 6.4 min (31 nm, N_MC_ = 45), 8 min (33 nm, N_MC_ = 60), and 10.7 min (35 nm, N_MC_ = 70), higher ILP values of 9.5, 11.7, and 14.5 nH∙m^2^∙kg_Fe_^−1^ are reached. To the best of our knowledge, such ILP are higher than all previously reported values for iron oxide nanomaterials, even superior to the value of 8.1 nH∙m^2^∙kg_Fe_^−1^ obtained very recently by Storozhuk et al.^49^ for 32 nm multi-core clusters synthetized by a seeded-growth polyol strategy. The magnetothermal behavior of the as-synthetized NFs is also investigated under various AMF conditions, for a frequency of 471 kHz and variable B amplitudes from 50 to 180 G (Fig. 3b, c). For residence times of 4, 4.6, and 5.8 min (13 nm $\le$ d_TEM_ $\le$ 24 nm, N_MC_ = 35), SLP increases linearly with the AMF amplitude (B). A behavior change is evidenced at higher residence times of 6.4 min (31 nm, N_MC_ = 45) and 10.7 min (35 nm, N_MC_ = 70) for which SLP varies as the square of the AMF amplitude (B^2^). Therefore, it suggests that the thermal losses subsequent to the AMF excitation are due to different relaxation processes, these being related to the magnetic properties’ evolution during the NFs formation. For τ_R_ $\le$ 5.8 min (N_MC_ = 35), the MHT efficiency is mostly caused by Néel relaxation while for τ_R_ $\ge$ 6.4 min (N_MC_ $\ge$ 45) Brownian fluctuations become predominant. To support such hypothesis, r_1_(*f* ) longitudinal NMR relaxivity profiles are measured. A strong evolution is observed depending on the residence times, going from the characteristic features for superparamagnetic nanoparticles (i.e., plateau at low frequency, broad peak around few MHz followed by drop of *R*_*1*_ at high frequency) to those for Brown dominated particles (i.e., quasi plateau at low frequency followed by *R*_*1*_ drop at high frequency)^50^. Importantly, this evolution is not monotonous. Up to 5.3 min, few changes occur. The peak is clearly visible and its position *f*(r_1,max_) is progressively slightly shifted to lower frequency (Fig. 3e). As shown in literature^51,52^, *f*(r_1,max_) shifts to lower frequency with the size of the particle, for a given polydispersity. From 5.8 min, the peak becomes suddenly barely visible and *f*(r_1,max_) seems to weakly change. To shed light on the underlying processes ruling these NMR relaxivity profiles, their characteristic features should be detailed. They consist in three components named the well-known Curie term, the average fluctuation of the Curie term and the transversal contribution^50^. The broad r_1_ peak (Fig. 3d) originating from the second contribution is linked to the Néel relaxation^53,54^. When the magnetic anisotropy increases, i.e., when the magnetic moment becomes blocked on the particles axis and the transversal contribution becomes predominant in the NMR ^1^H relaxation processes masking this peak^50,55^. Taken together, both MHT and NMR relaxometry results evidence the behavior modification in terms of magnetic spin relaxation, going from Néel-mode dominant NFs to Brownian-mode dominant NFs after τ_switch_ = 5.8 min.

### The crucial role of nanoclusters mosaicity

**Fig. 4:**
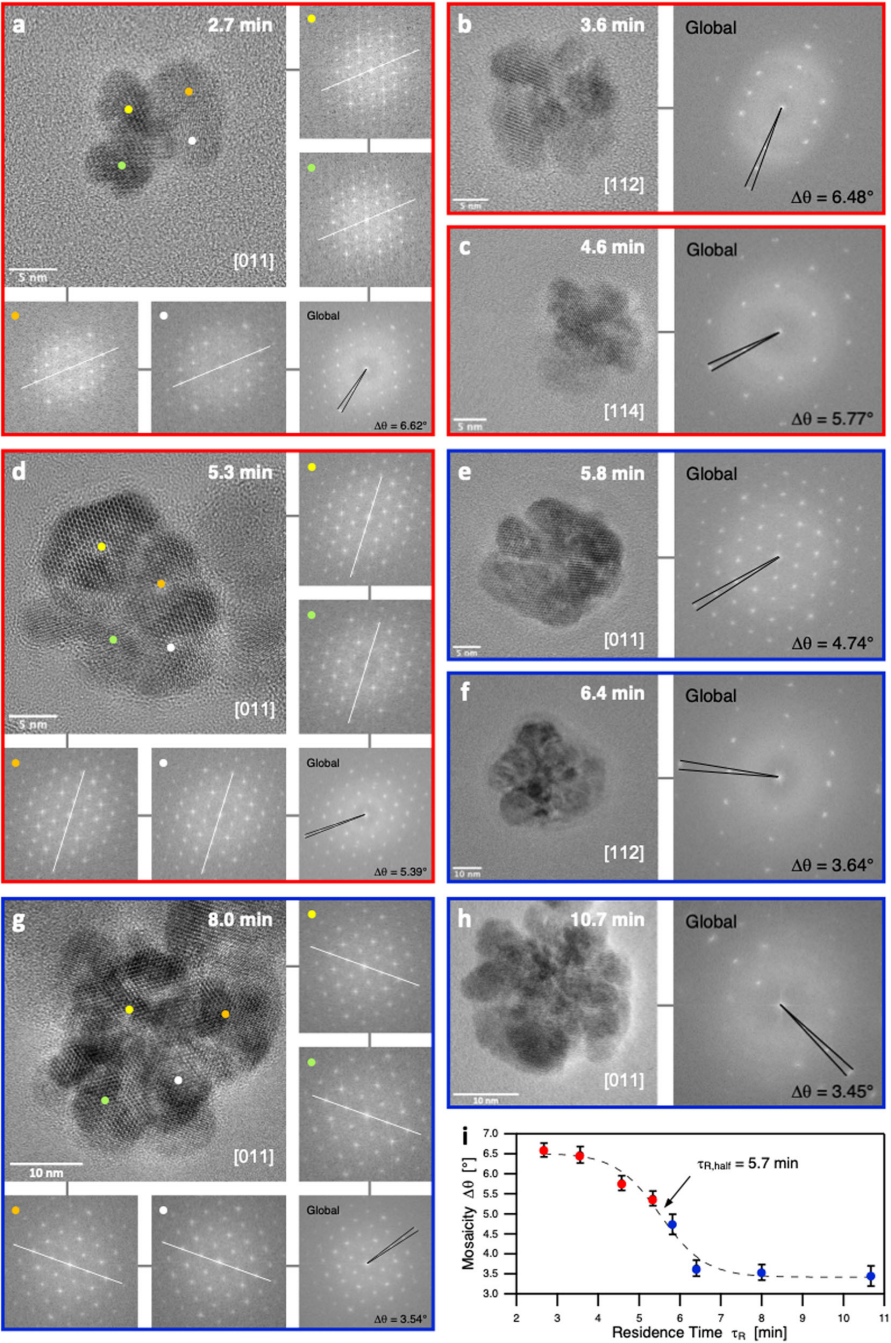
Fine-structure analysis of core disorders. **a**–**h** HR-TEM micrographs of the NFs and their corresponding FFT for mosaicity measurements (Δ*θ*) during growth (red: **a**, **b**, **c**, **d**) and aggregation (blue: **e**, **f**, **g**, **h**) stages. **i** Mosaicity as a function of residence time. Error bars correspond to the standard deviation obtained from the measurements of mosaicity on 15 different isolated NFs for each residence times. White solid lines: eye-guide for an easier comparison of the local FFT; black solid lines: angular measurements of the mosaicity values.

In the case of molecular magnets, numerical simulations and experiments have shown that the magnetic coupling (*E*_*i,j*_) between two atom centers *i* and *j* is described by:

1 $${E}_{i,j}=-{J}_{i,j}\cdot {S}_{i}\cdot {S}_{j}\cdot \cos ({\theta}_{i,j})$$

where *J*_*i,j*_ is the coupling constant, *S*_*i*_ and *S*_*j*_ the magnetic spins of centers *i* and *j*, and *θ*_*i,j*_ the angle between the two atom magnetic spins. As *θ*_*i,j*_ regulates magnetic properties of molecular magnets, HR-TEM were performed to evaluate the misalignment of the different cores within a single NF. The angular dispersion, also called mosaicity, is a measure of the spread of the crystal plane orientations that can be obtained from the angular sprawl of diffraction peaks on the fast Fourier transform (FFT) of HR-TEM images for each residence time (Fig. 4a–h). In some cases, FFT are measured on four different areas inside the same nanoassembly (Fig. 4a, d, g). The four distinct FFT show very similar signatures and are superimposable with the global FFT of the whole nanoassembly. Such an observation evidences the highly monocrystalline character of the as-synthetized NFs. Strikingly, the evolution of mosaicity (Δ*θ*) with residence time follows a sigmoid decreasing function characterized by τ_R,half_ = 5.7 min (Fig. 4i), close to all previously determined characteristic times associated to nanostructural, magnetic, and optical property switches. Based on these observations, we consequently draw the following mechanism: the clusters generated after the nucleation and attachment steps (Fig. 1f) are attached with Δ*θ*_max_ = 6.5° probably driven by an oriented auto-assembling process^56^. During the subsequent growth stage (Fig. 1f), while each magnetic core grows by molecular diffusion, the crystalline cores continue progressively to align with each other. The oriented attachment continues also during the final aggregation stage (Fig. 1f), where the fusion of one NF to other leads to an angular defect of only Δ*θ*_min_ = 3.5°.

**Fig. 5:**
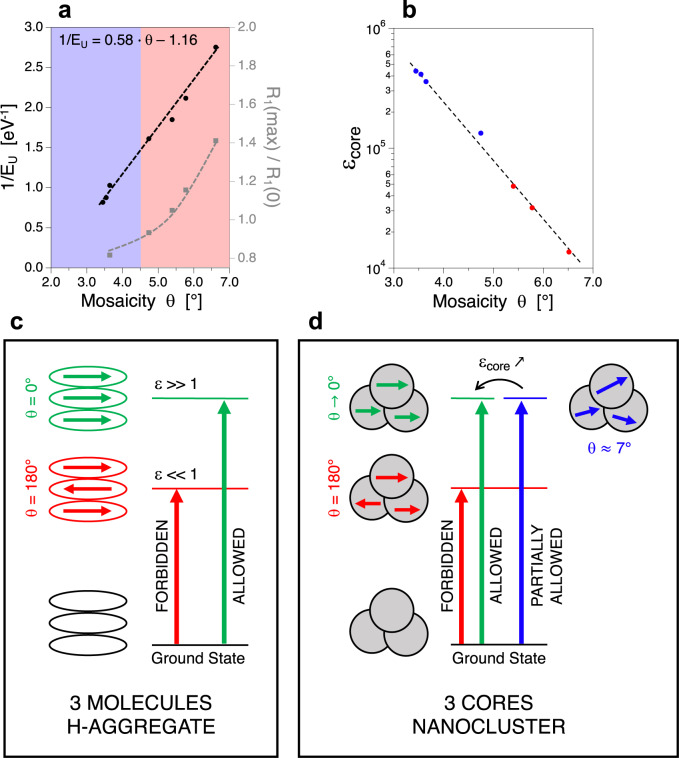
Impact of mosaicity on the optical properties. **a** Defect-characteristic 1/E_U_ values (black circles) and Néel contribution (R_1_(max)/R_1_(0), gray squares) as a function of mosaicity. **b** Evolution of the extinction coefficient of one core (ε_core_ = ε_NP_/N_MC_) at 1064 nm with mosaicity. **c** Description of the optical theory for excitonic absorption in molecular H-aggregates. **d** Analogy of the molecular excitonic theory in the case of multi-core nanoclusters such as NFs.

According to molecular magnetism theory (Eq. 1), important *θ*_*i,j*_ variations triggers major changes in the magnetic coupling: such an effect is observed in the case of NFs for which mosaicity variations are responsible for both M_S_ and ILP changes. Finally, both 1/E_U_ values and R_1_(max)/R_1_(0) values, characteristic of the Néel contribution in magnetic spin relaxation processes^31^, are plotted against mosaicity (Fig. 5a). Disorder effects at core boundaries should lead to changes in E_U_ values, as previously discussed. The linear increase in 1/E_U_ = *f*(Δ*θ*) also suggests that a crystal with a mosaicity of 2° should present an infinite E_U_ value, so that core boundaries defects become neglectable for Δ*θ* $\le$ 2°. Moreover, the increase in Brownian spin relaxation with residence times evidenced by relaxometry measurements can be ascribed to the diminution of mosaicity. As the alignment of the cores goes from to Δ*θ*_max_ = 6.5° to Δ*θ*_min_ = 3.5°, the nanoassembly becomes more coherent and NFs tend to behave as one single big rigid magnetic dipole rather than a cluster composed of several cores^57^. Concerning optical properties, a similar analogy can be established between molecular theories and NFs optical features. The excitonic theory for supramolecular assemblies predicts that the angle *θ* between electronic absorption momentum of aggregated molecules is the geometrical parameter that controls electronic transitions^58^. In the case of molecular H-aggregates, which consist in a molecular π-stacking, electronic transitions become more and more symmetrically allowed when *θ* decreases from 180° (antiparallel configuration) to 0° (parallel configuration). Such phenomenon can be ascribed by the evolution of extinction coefficient values, signature of an electronic transition. In the case of NFs, a high increase in ε_core_ is observed when the mosaicity Δ*θ* decreases (Fig. 5b). Assuming that the direction of core absorption momentum is conditioned by the crystallographic core orientations, the evolution of ε_core_ with Δ*θ* reflects that the electronic transition around 1064 nm (IVCT) is more allowed when cores tend to reach a parallel configuration. As a result, we can conclude that flower-like nanoclusters obey to the excitonic theory for H-aggregates (Fig. 5c, d), where each core acts as a molecular brick whose orientation conditions the electronic transitions.

### Conclusion

The correlation of NFs formation mechanism at the nanoscale with their magneto-optical properties and their fine high-resolved structures evidences the crucial role of mosaicity. This latter is found to regulate the static and dynamic magnetic properties of the molecular assemblies impacting their function by hyperthermia. Moreover, the nanoclusters can be described as a supramolecular optical aggregate where each core acts as a molecular brick whose orientation regulates the optical properties, as predicted by the molecular excitonic theory for H-aggregates. We believe that these results will have direct impact in the field materials science providing us with a new bottom-up concept based on manipulation of mosaicity by chemical synthesis conditions in order to program their individual or collective properties for optimized functions.

## Experimental section

### Materials

N-methyldiethanolamine (NMDEA, >99%), diethylene glycol (DEG, >99%), iron(III) chloride hexahydrate (FeCl_3_∙6H_2_O, 99%), iron(II) chloride tetrahydrate (FeCl_2_∙4H_2_O, 99%) are purchased from Merck (Darmstadt, Germany). Sodium hydroxide pellets (NaOH, 99%), hydrochloric acid (HCl, 37%), ethanol (96%), nitric acid (HNO_3_, 68%), acetone (>99%), and diethyl ether (Et_2_O, 100%) are purchased from VWR International (Rosny-sous-Bois, France). All chemicals are used without further purification. Analytical HPLC pump ECP2000 is purchased from ECOM (Prague, Czech Republic).

### Millifluidic flow system

Stainless steel millifluidic channels (inner diameter: 0.040″, outer diameter: 1/16″) are purchased from Cluzeau Info Lab (Sainte-Foy-la-Grande, France). Proportional-integral-differential (PID) controllers (77 mm × 35 mm, 220 V, −200 to 600 °C), Pt100 temperature probes, heating cartridges (16 mm × 200 mm, 1 kW, 220 V), electrical fuses (16A and 64A), on/off switching buttons and electrical cables (1.3 mm^2^ and 2.1 mm^2^) are from Radio Spare Pro™ (Beauvais, France).

### Flow synthesis procedure

All the iron oxide NFs presented in this work are synthetized using a previously described millifluidic system by a modified polyol route^32^. The flow system is filled with DEG using a flow rate of 5 mL∙min^−1^ during 4 min. The PID control boxes are then set to a temperature of 220 °C and the flow rate is decreased to 1 mL∙min^−1^. Once the thermal equilibrium is reached (about 5 min), the reactive media is used as the inlet solution and is injected at the desired flow rate. After a time of t = 1.5 τ_R_, the collecting flask is replaced by a new one to collect only the products of the reaction (without collecting the initial DEG or eventual impurities). Once a sufficient volume of crude product is obtained, the collecting flask is replaced by another one. The PID control boxes are set to 20 °C and a DEG flow of 2 mL∙min^−1^ is injected to clean the inside of the millifluidic channel while the temperature decreases. Once the system reaches ambient temperature, 200 mL of water are injected at a high flow rate of 10 mL∙min^−1^ to remove any remaining impurities. Finally, the HPLC pump, each PID control box and the general electrical control box are switched off.

### Ex situ kinetic studies

At the end of the millifluidic device, the crude product is collected in a flask which is placed in a cooling bath in order to quench the reaction yielding to the formation of NFs. The crude product is then washed and characterized by several methods. With such a process, ex situ kinetic studies are both easy and relevant as the reaction is stopped at the outlet of the system.

### NFs characterization

The total iron concentrations of NFs suspensions are measured by atomic absorption spectroscopy (AAS, PinAAcle 500, Perkin Elmer) degrading the samples in concentrated HCl (37%) before a dilution in HNO_3_ (2%). NFs are imaged using a JEOL-1011 transmission electron microscope operating at 100 kV. Size distributions are determined thanks to Image J software by measuring manually 300 NFs on at least three different images. The resulting histograms are modelized by log-normal laws using Igor Pro 7 software to determine the mean physical diameter and the polydispersity of each sample. High-resolution TEM is performed on a JEOL 2010 operating at 200 kV. Magnetization curves (for Langevin models) are measured using a home-made vibrating sample magnetometer in the range 0–800 kA/m. Magnetization hysteresis loops (for M_S_ determination) are measured on a MPMS-XL7 Quantum Design SQUID in the range −20 kOe to 20 kOe.

### Determination of the number of magnetic cores per nanoassembly

The size of each magnetic core (d_m_) are determined thanks to M = f(H) curves modeled by a log-normal weighted Langevin law. As the physical diameter (d_TEM_) of the whole nanoassembly is known (by TEM measurements), assuming that both magnetic cores and nanoassemblies are spherical, one can obtain the number of magnetic cores (N_MC_) per nanoassembly: N_MC_ = (d_TEM_/d_m_)^3^.

### Magnetic hyperthermia

The experiments are carried out in 0.5 mL Eppendorf® containing 50 μL of the NPs suspensions excited for 600 s by an alternating magnetic field generator device (DM3, NanoScale Biomagnetics) operating at a frequency of 471 kHz with amplitudes ranging from 50 G to 180 G Temperature increase are measured by an infrared camera FLIR SC7000.

### NMR relaxometry

The measurements of the water ^1^H longitudinal relaxation rates R_1_ = 1/T_1_ (and the related relaxivity r_1_ = R_1_/[Fe]) have been carried out using Stelar SpinMaster relaxometer, in the 10 kHz to 30 MHz frequency range (^1^H frequency). A pre-polarized sequence has been used from 10 kHz to 10 MHz and a non-polarized sequence from 10 MHz to 30 MHz^59^. The samples put in glass tubes 10 mm external in diameter and 40 mm in length closed by a silicon cap, were thermostated at 298 K using regulated air flux.

### X-ray scattering and absorption

Small angle X-ray scattering is performed at Synchrotron SOLEIL on the SWING line in 1 mm diameter glass capillary. X-ray absorption near edge spectroscopy at Fe K-edge is performed at Synchtoron SOLEIL on the ROCK line in 1 mm diameter glass capillary, using a Si(111) monochromator.

### Optical characterization

UV-Vis-NIR spectra (400–1100 nm) are recorded at room temperature in a 1 cm quartz cuvette using an Avantes spectrophotometric set-up composed of an AvaLight-DHc lamp connected by optical fibers to a StarLine AvaSpec UV/Vis detector and to a NIRLine AvaSpec-NIR256-1.7 NIR detector.

## Supplementary information

Supplementary Information

## Data Availability

A Supplementary Information file is available together with this manuscript. It includes a global description of the millifluidic system, TEM of figures corresponding to the different rresidence times, size distribution histograms analysis, magnetization curves, SAXS analysis, magnetization curves, XANES analysis, UV-Vis-NIR absorption spectra, Beer Lambert plots, Tauc plots, Urbach plots and temperature elevation curves obtained in MHT. The data that support the findings of this study are available from the corresponding author A.A.-.H.
