# Supplementary material for: Angular orientation between the cores of iron oxide nanoclusters controls their magneto–optical properties and magnetic heating functions
Source: Commun Chem. 2022 Dec 2;5:164. doi: 10.1038/s42004-022-00787-0 (PMC9814453; doi:10.1038/s42004-022-00787-0)
Supplement: Supplementary file 1 — Supplementary Information [file 42004_2022_787_MOESM1_ESM.pdf]

SUPPLEMENTARY INFORMATION

Angular orientation between the cores of iron oxide nanoclusters controls their magneto–optical properties and magnetic heating functions

*Enzo Bertuit[†], Nicolas Menguy[#], Claire Wilhelm [§], Anne-Laure Rollet[†] and Ali Abou-Hassan[†,⊥,]**

[†]Sorbonne Université, UMR CNRS 8234, PHysico-chimie des Électrolytes et Nanosystèmes InterfaciauX (PHENIX), F-75005 Paris, France

[#]Sorbonne Université, UMR 7590 CNRS – Sorbonne Université – IRD-MNHN, Institut de Minéralogie, de Physique des Matériaux et de Cosmochimie (IMPMC), Case 115, 4 Place Jussieu, 75252 Paris Cedex 5, France

[§]PSL Research University − Sorbonne Université – CNRS, UMR168, Laboratoire Physico Chimie Curie, Institut Curie, 75005 Paris, France

[⊥]Institut Universitaire de France (IUF), 75231 Paris Cedex 05, France

*Authors to whom correspondence should be addressed:

ali.abou_hassan@sorbonne-universite.fr

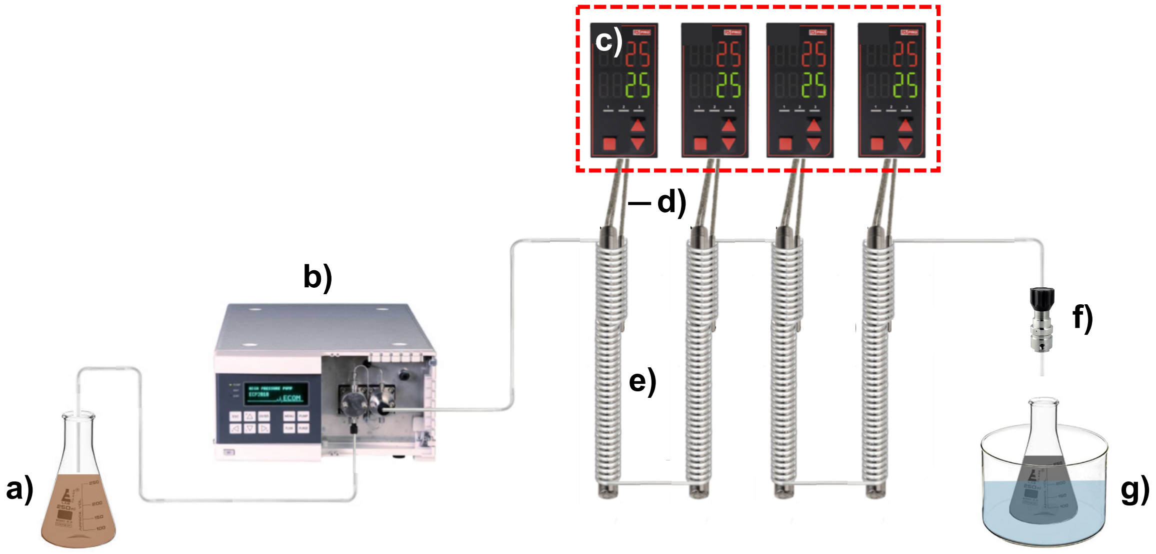

Figure S1. Global description of the multi-parametric millifluidic device. a–g) Schematic representation of the different elements composing the system with a) the initial reactive media; b) the HPLC pump; c) the proportional-integral-differential control boxes; d) the temperature probes; e) the heating cartridges; f) the back-pressure regulator and g) the collecting flask.

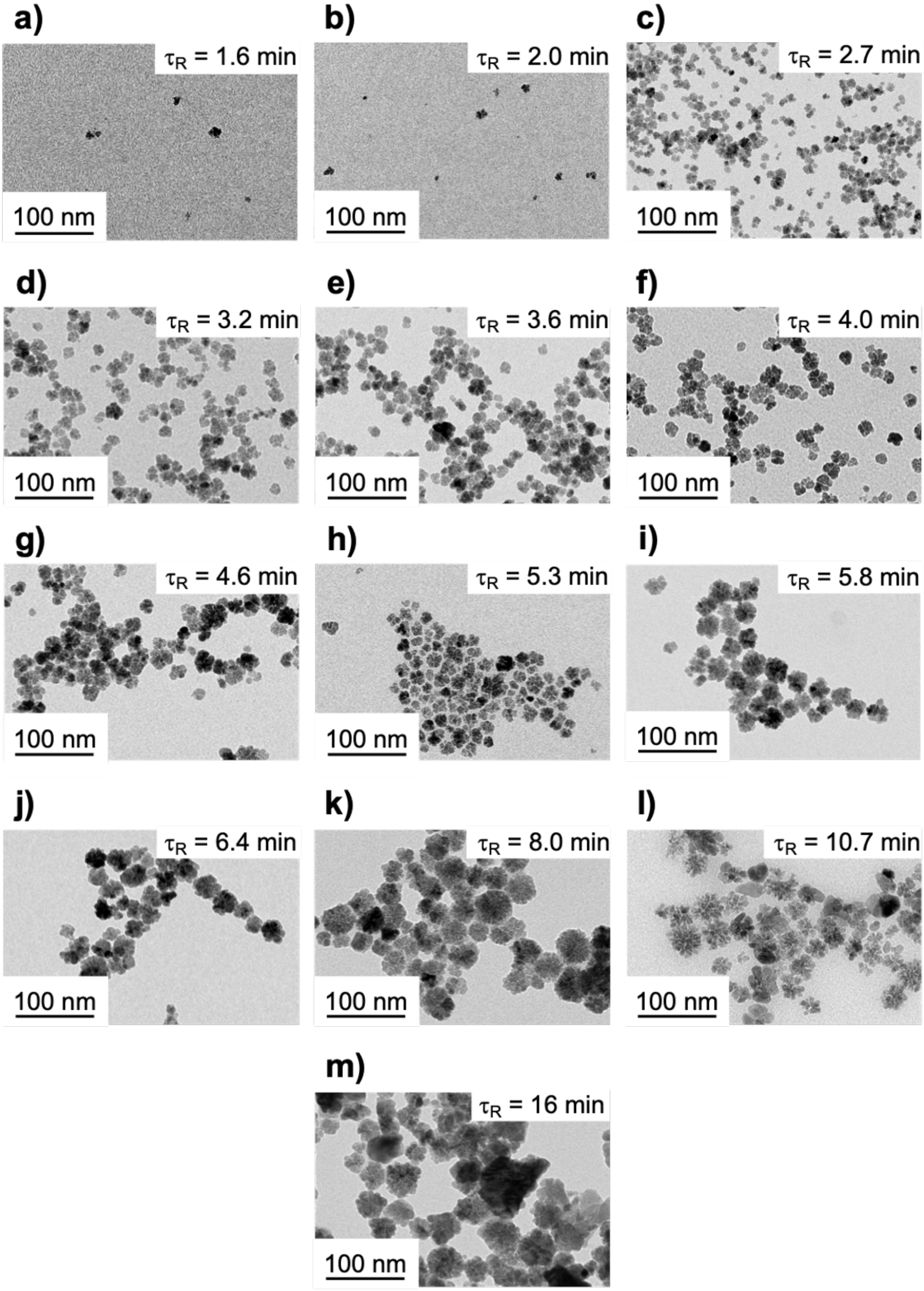

Figure S2. Representative TEM micrographs of Fe_3O_4 nanoflowers obtained for different residence times ranging from 1.6 min to 16 min. All scale bars are 100 nm.

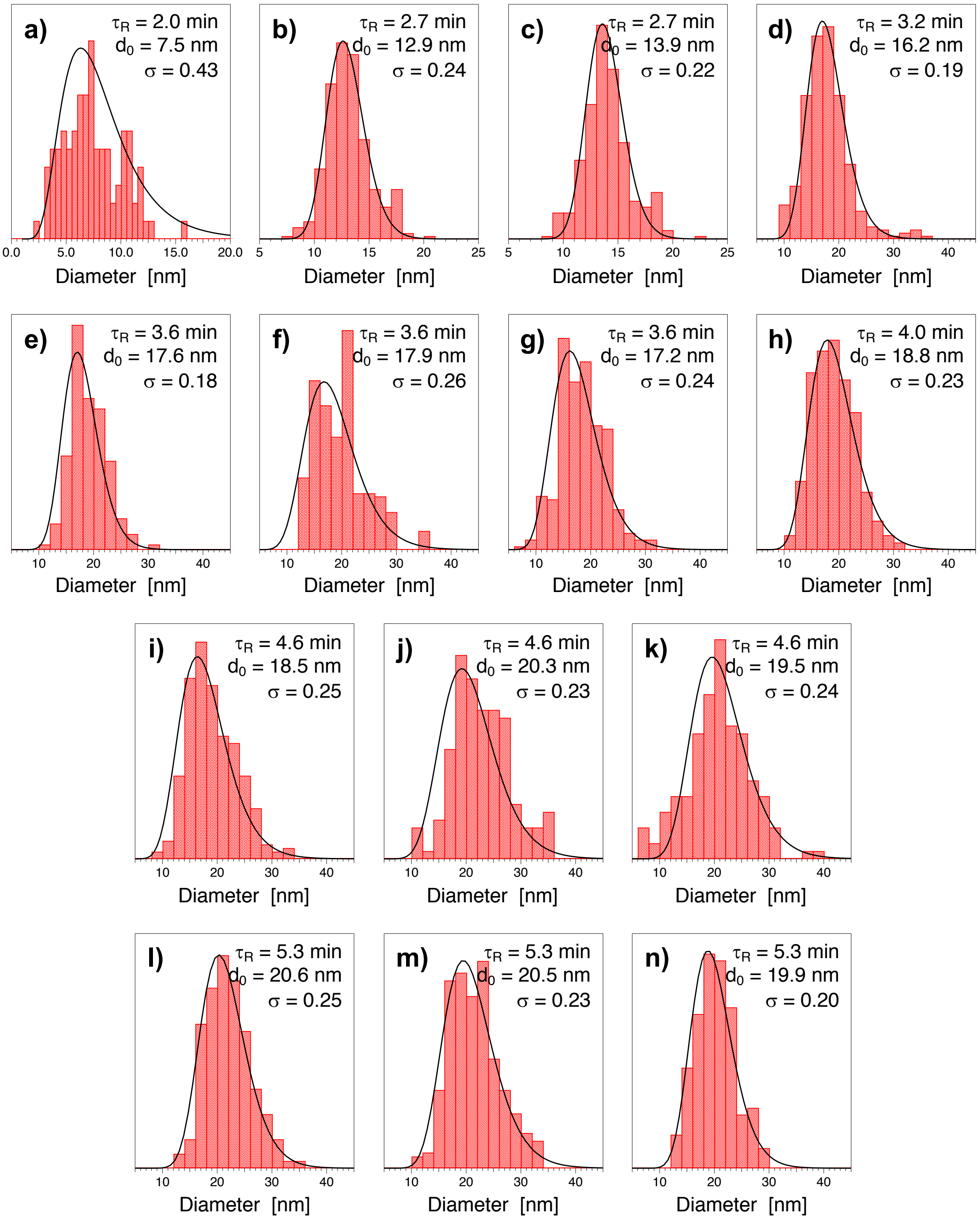

Figure S3. Size-distribution histograms obtained from TEM micrographs analyses of Fe_3O_4 NFs for different residence times ranging from 2.0 min to 5.3 min. Syntheses were repeated several times for the same residence time to evidence the good reproducibility.

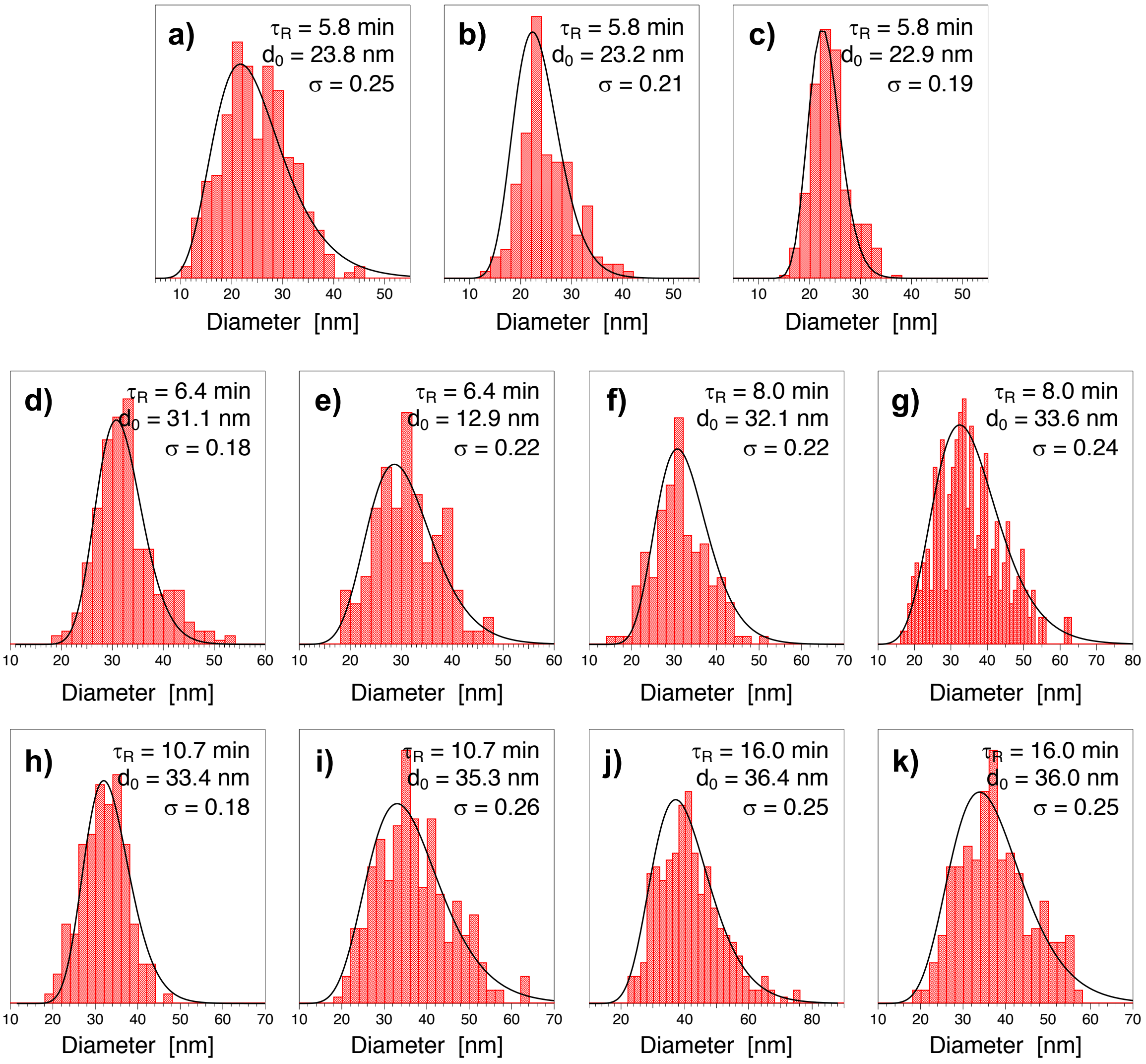

Figure S4. Size-distribution histograms obtained from TEM micrographs analyses of Fe_3O_4 NFs for different residence times ranging from 5.8 min to 16 min. Syntheses were repeated several times for the same residence time to evidence the good reproducibility.

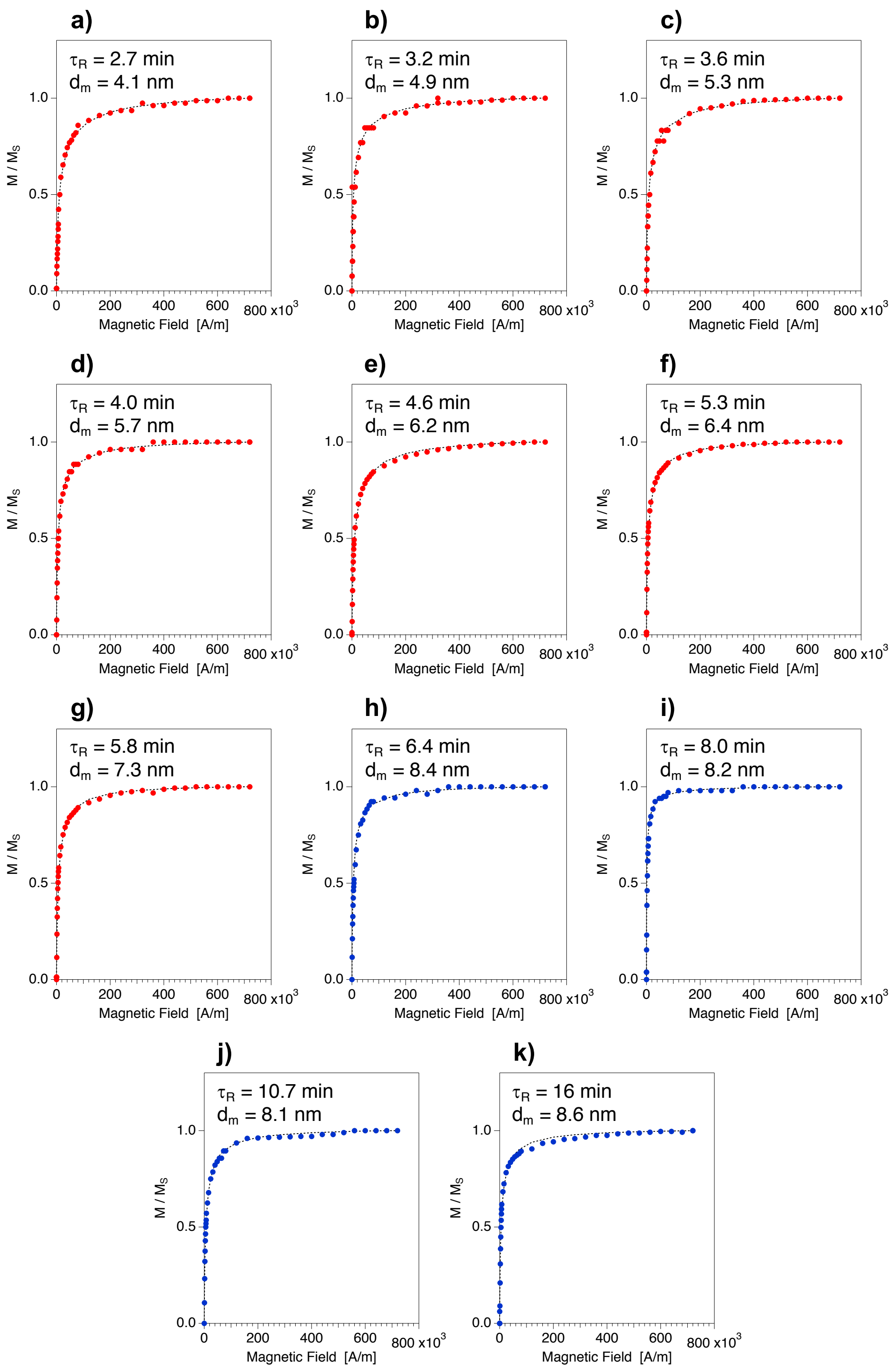

Figure S5. Normalized magnetization curves recorded on colloidal suspensions at 300K. Black solid lines: model by a log-normal weighted Langevin law giving magnetic core sizes (d_m).

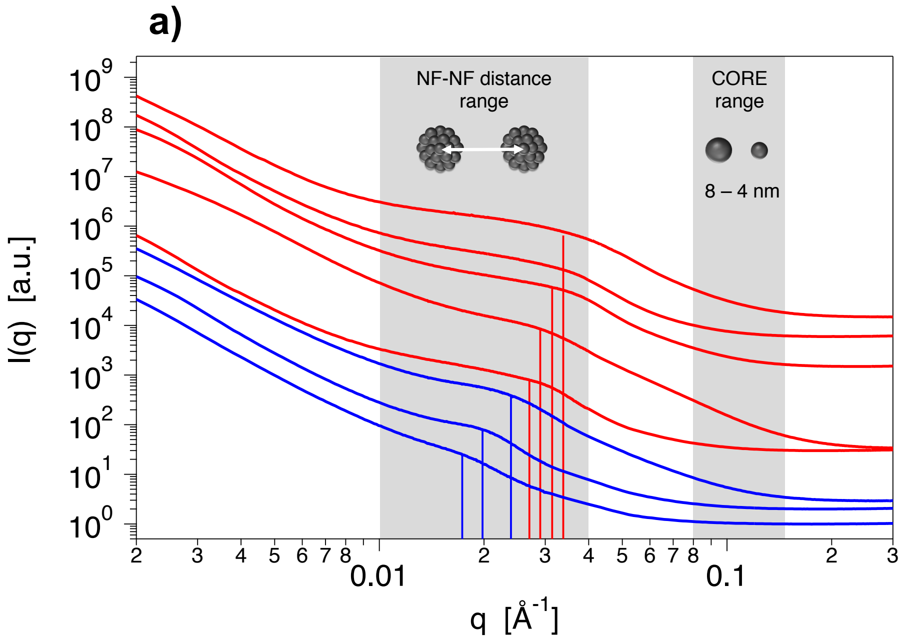

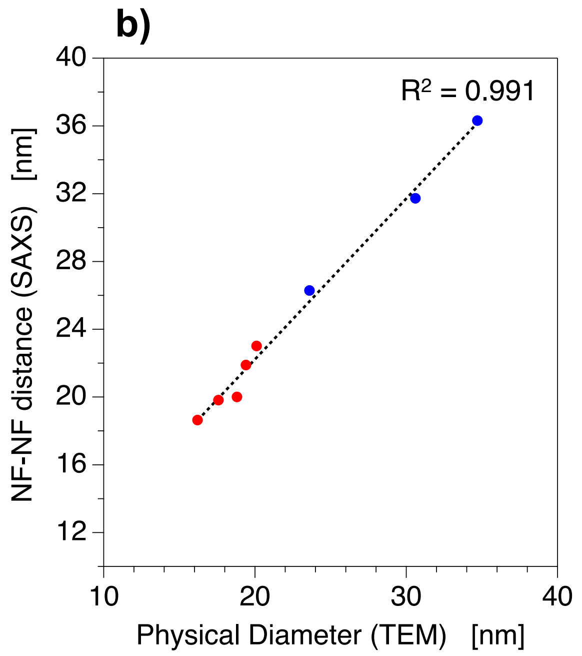

Figure S6. a) Small angle X-ray scattering patterns for residence times of 3.2, 3.6, 4, 4.6, 5.3, 5.8, 6.4 and 10.7 min (from top to bottom) evidencing a clear NF-NF interaction peak while no peak is observed in the 4-8 nm core size region. b) Linear correlation between NF-NF distances and physical NFs diameter meaning that NFs are aggregated into big clusters of several NFs in the crude final product (polyol solvents).

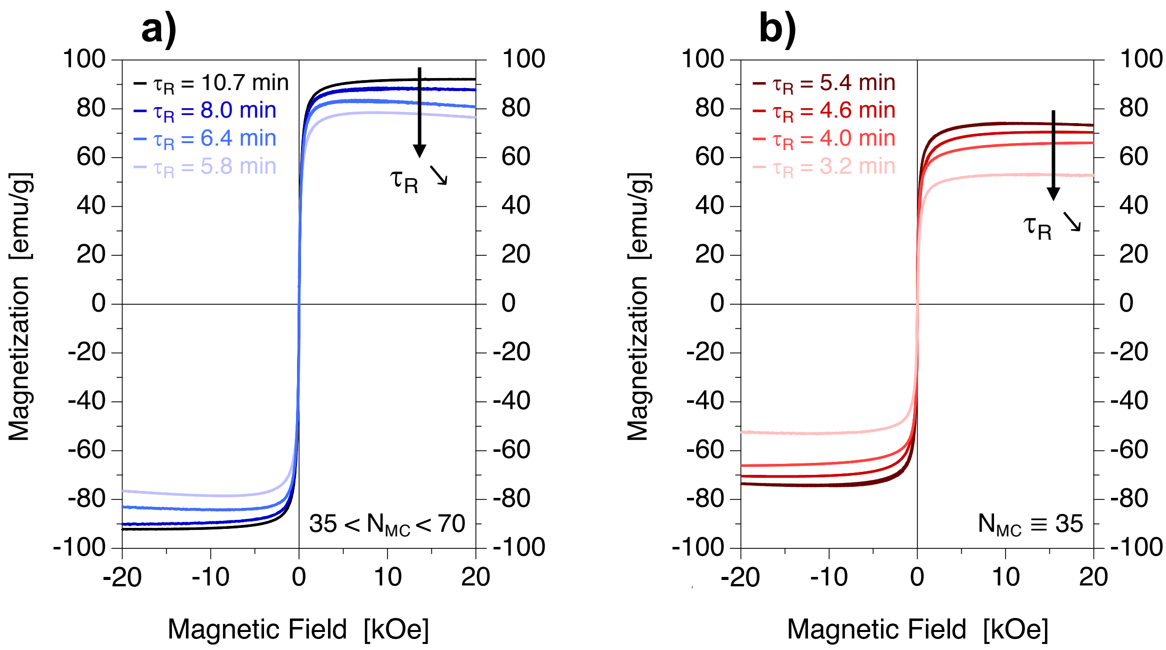

Figure S7. Magnetization curves recorded on colloidal suspensions at 300K using a SQUID device to obtain absolute values in emu/g for a) NFs during the aggregation stage and b) NFs during the growth stage.

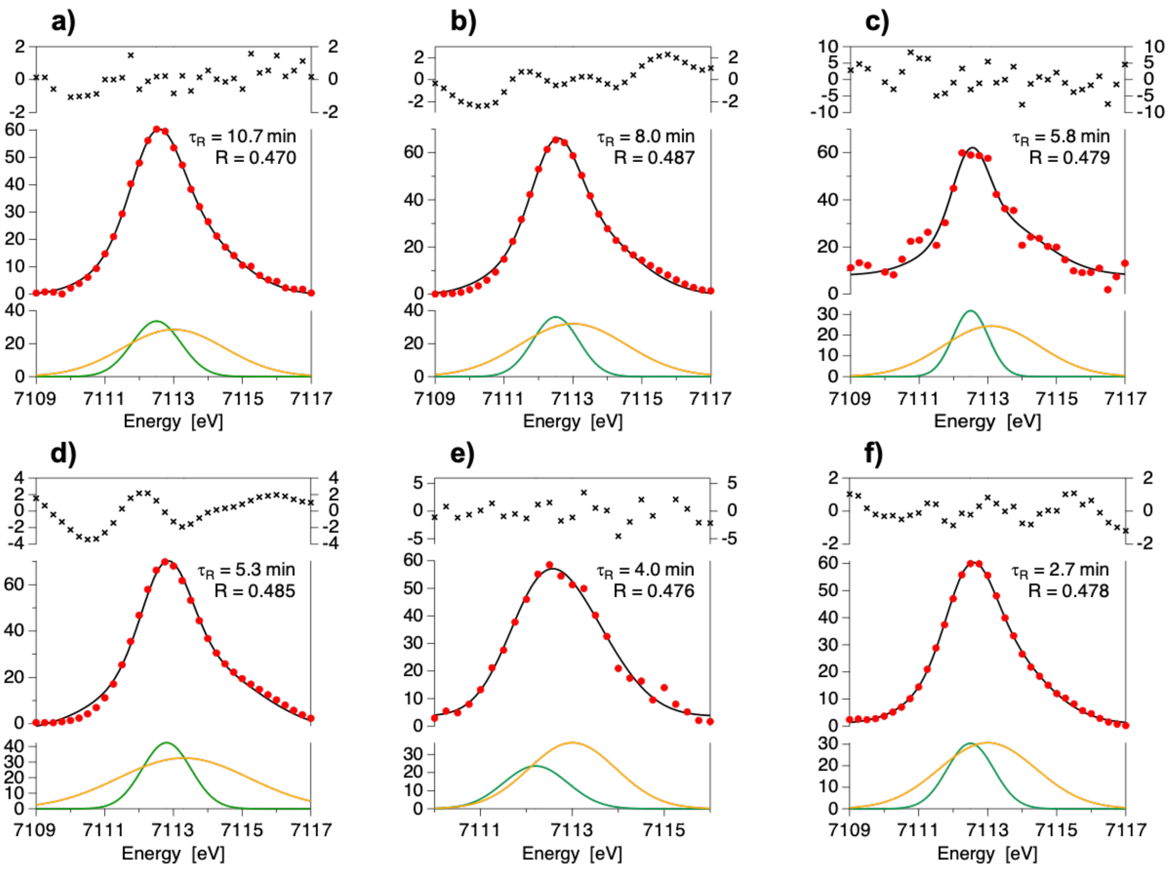

g)

τ_R (min)	Fe(III) contribution		Fe(II) contribution		R = Fe(II) / Fe(III)	
	Area	Error	Area	Error	Value	Error
10.7	0.10665	0.0044911	0.050089	0.0023873	0.470	0.03
8.0	0.12404	0.0077019	0.060388	0.0036811	0.487	0.04
5.8	0.085878	0.0018592	0.041146	0.0078129	0.479	0.09
5.3	0.155555	0.0017995	0.075389	0.0051696	0.485	0.03
4.0	0.087152	0.0070925	0.041512	0.0046771	0.476	0.07
2.7	0.10793	0.0030463	0.051664	0.001741	0.479	0.02

Figure S8. a–f) XANES analyses for stoichiometry R = Fe^{II}/Fe^{III} determination. Red points: pre-edge peak experimental data, black solid lines: 2–peaks models, black crosses: residuals, green solid lines: gaussian iron(II) contribution (7112.5 eV), orange solid lines: gaussian iron(III) contribution (7113 eV). g) Summary of the fit results.

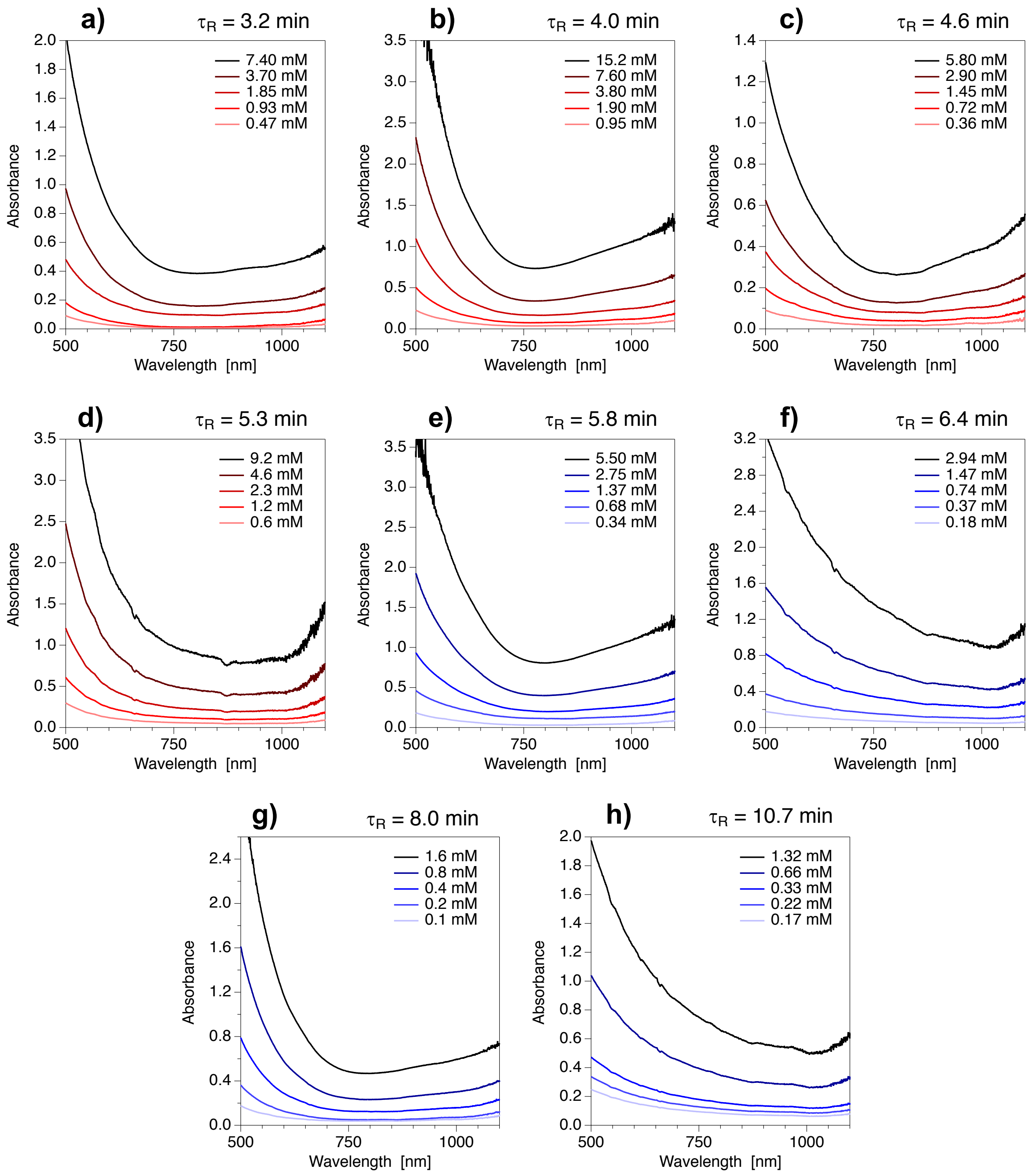

Figure S9. UV-Vis-NIR absorption spectra recorded on colloidal suspensions with different iron concentrations for each residence time. The iron concentrations were determined by AAS.

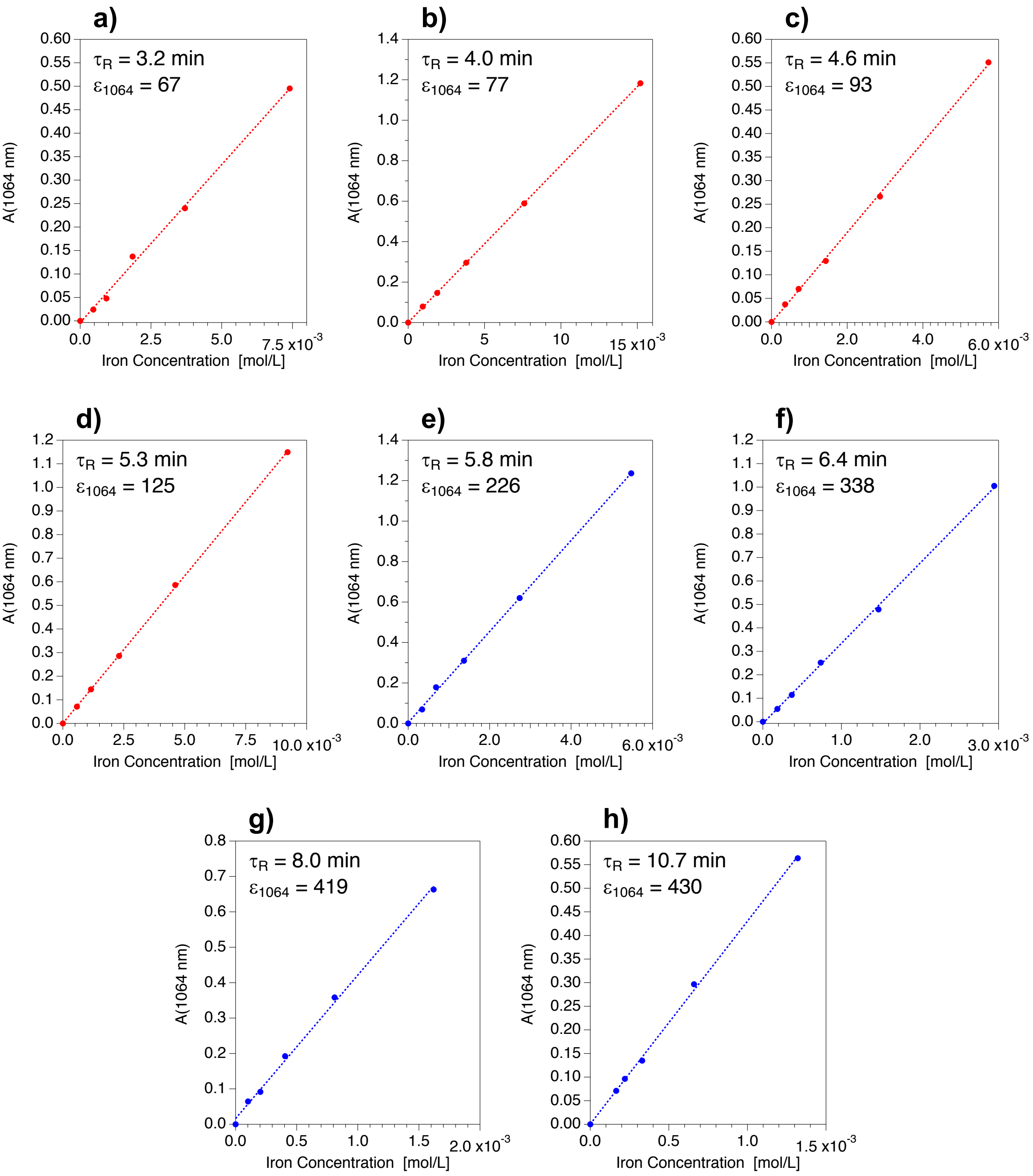

Figure S10. Beer-Lambert plots at 1064 nm obtained from UV-Vis-NIR spectra, giving the values of extinction coefficient ε(1064) in $L{\cdot}mol_{Fe}^{-1}{\cdot}cm^{-1}$ for different residence times.

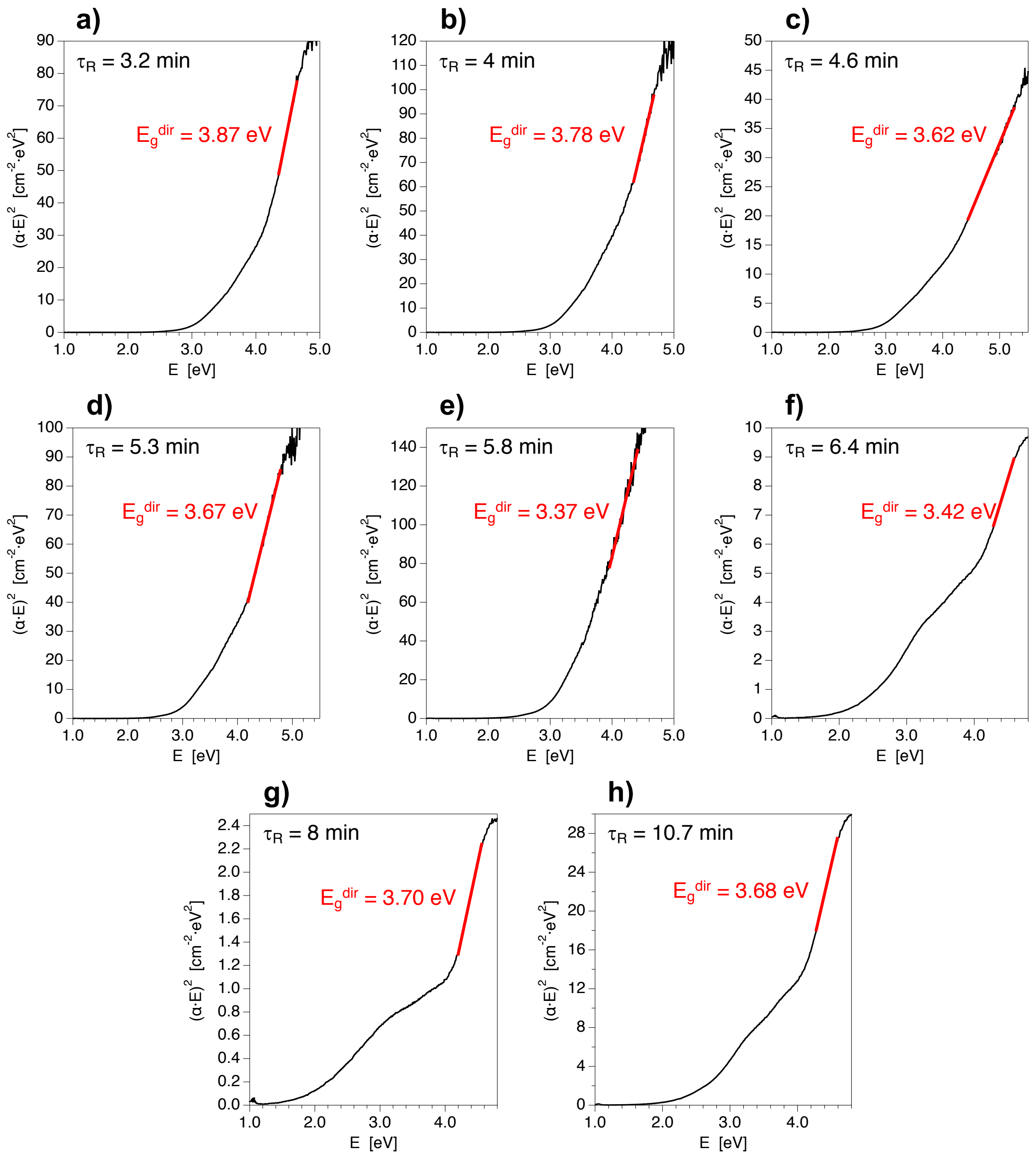

Figure S11. Tauc plots for the determination of direct band gap energy (E_g^{dir}), where *E* stands for the photon energy and α is the absorption coefficient.

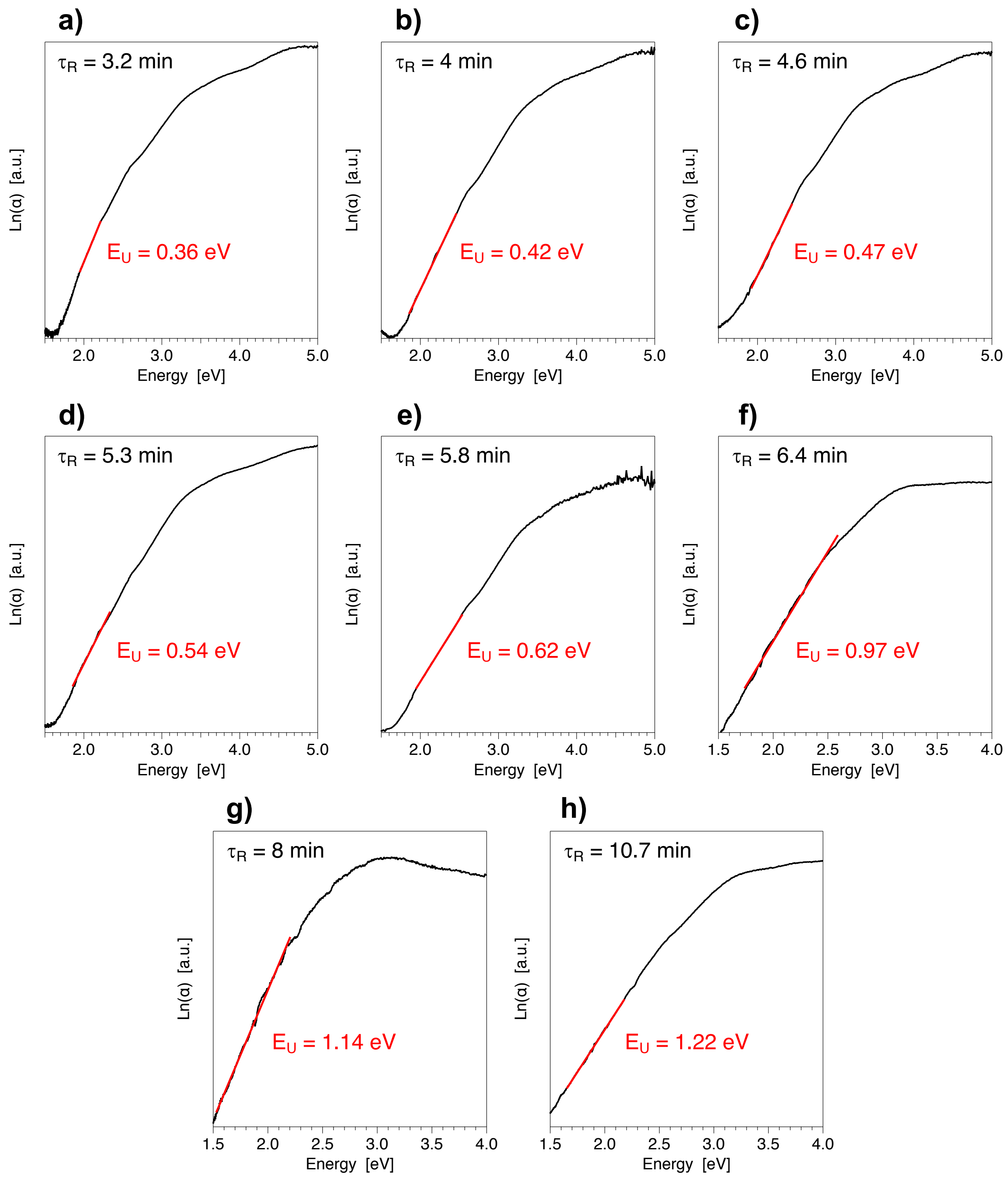

Figure S12. Urbach plots for the determination of defect-characteristic Urbach energy values, where α is the absorption coefficient.

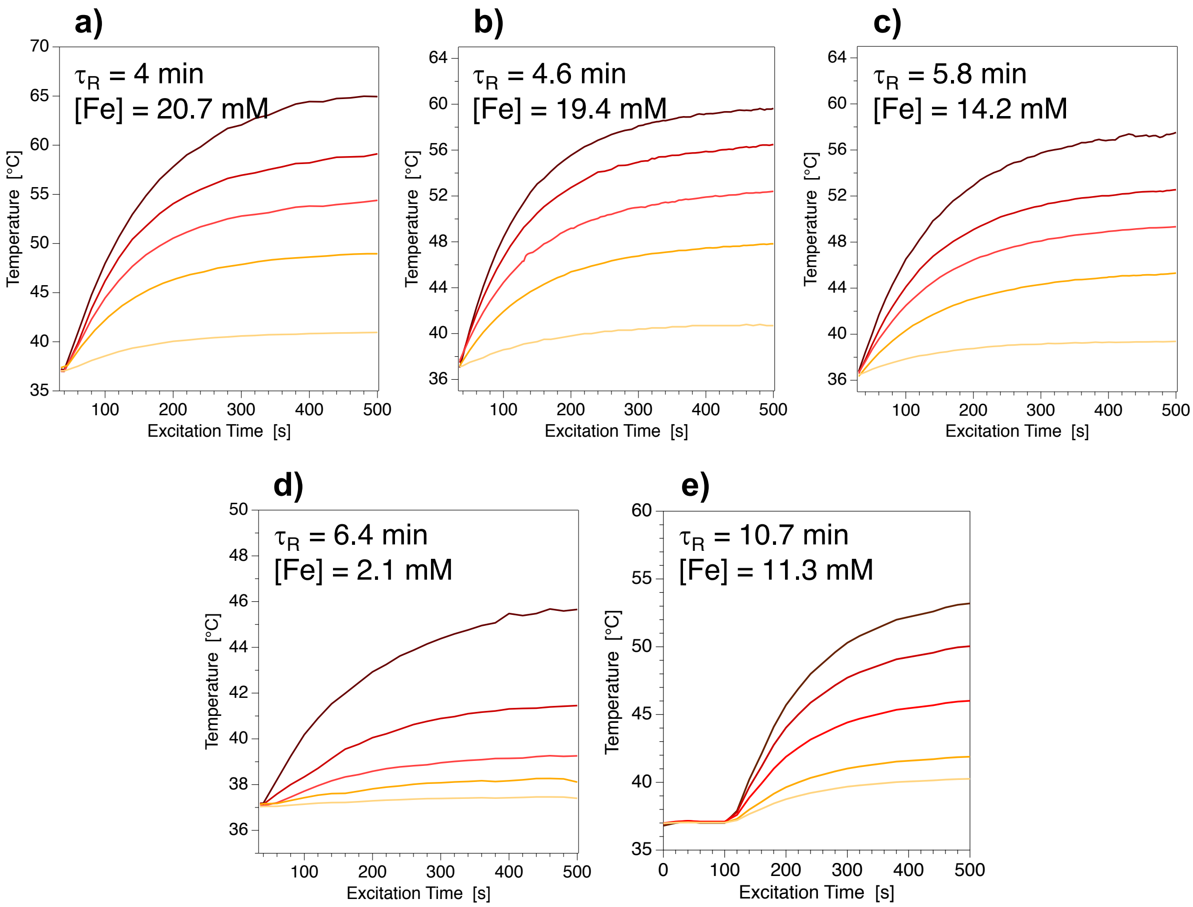

Figure S13. Temperature elevation curves recorded during magnetic hyperthermia experiments for an AMF operating at 471 kHz with different strengths of 180 G (brown), 150 G (dark red), 120 G (red), 90 G (orange) and 50 G (yellow). The SLP are determined using the linear regime of the curves in the first 30 seconds of temperature increase. The iron concentrations were determined by AAS.
